# Supplementary material for: Molecular Analysis in a Glioblastoma Cohort—Results of a Prospective Analysis
Source: J Pers Med. 2022 Apr 26;12(5):685. doi: 10.3390/jpm12050685 (PMC9148107; doi:10.3390/jpm12050685)
Supplement: Supplementary file 1 [file jpm-12-00685-s001.zip › jpm-1660032-supplementary.pdf]

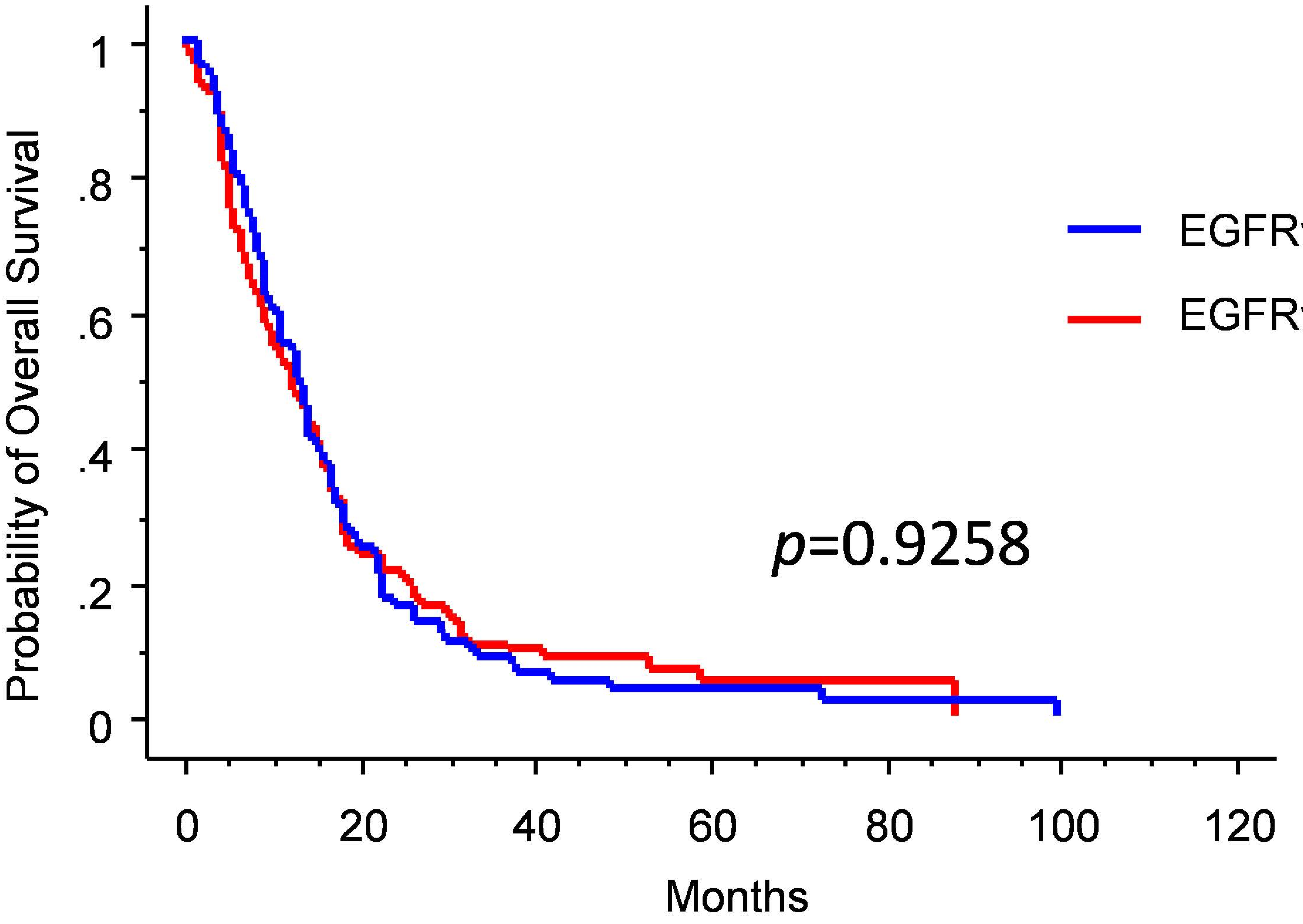

Supplementary Figure S1. Kaplan-Meier survival curves for EGFRvIII expression in patients with confirmed IDH-wildtype status.
